# Determination and prediction of amino acid digestibility in brown rice for growing-finishing pigs

**DOI:** 10.5713/ab.23.0455

**Published:** 2024-04-24

**Authors:** Qing Ouyang, Rui Li, Ganyi Feng, Gaifeng Hou, Xianji Jiang, Xiaojie Liu, Hui Tang, Ciming Long, Jie Yin, Yulong Yin

**Affiliations:** 1 College of Animal Science and Technology, Hunan Agricultural University, Hunan Co-Innovation Center of Animal Production Safety, Changsha 410128, China; 2 Key Laboratory of Agro-Ecological Processes in Subtropical Region, National Engineering Laboratory for Poultry Breeding Pollution Control and Resource Technology, Key Laboratory of Animal Nutritional Physiology and Metabolic Process, Institute of Subtropical Agriculture, Chinese Academy of Sciences, Changsha 410125, China

**Keywords:** Amino Acids Digestibility, Brown Rice, Pigs, Prediction Model

## Abstract

**Objective:**

The experiment aimed to determine the standardized ileal digestibility (SID) of crude protein (CP) and amino acids (AA) in 10 brown rice samples fed to pigs, and to construct predictive models for SID of CP and AA based on the physical characteristics and chemical composition of brown rice.

**Methods:**

Twenty-two cannulated pigs (initial body weight: 42.0±1.2 kg) were assigned to a replicated 11×3 incomplete Latin square design, including an N-free diet and 10 brown rice diets. Each period included 5 d adaptation and 2 d ileal digesta collection. Chromic oxide was added at 0.3% to all the diets as an indigestible marker for calculating the ileal CP and AA digestibility.

**Results:**

The coefficients of variation of all detected indices for physical characteristics and chemical composition, except for bulk weight, dry matter (DM) and gross energy, in 10 brown rice samples were greater than 10%. The SID of CP, lysine (Lys), methionine, threonine (Thr), and tryptophan (Trp) in brown rice was 77.2% (62.6% to 85.5%), 87.5% (80.3% to 94.3%), 89.2% (78.9% to 98.9%), 55.4% (46.1% to 67.6%) and 92.5% (86.3% to 96.3%), respectively. The best prediction equations for the SID of CP, Lys, Thr, and Trp were as following, SID_CP_ = −664.181+8.484×DM (R^2^ = 0.40), SID_Lys_ = 53.126+6.031×ether extract (EE)+0.893×thousand-kernel volume (R^2^ = 0.66), SID_Thr_ = 39.916+7.843×EE (R^2^ = 0.41), and SID_Trp_ = −361.588+4.891×DM+0.387×total starch (R^2^ = 0.85).

**Conclusion:**

Overall, a great variation exists among 10 sources of brown rice, and the thousand-grain volume, DM, EE, and total starch can be used as the key predictors for SID of CP and AA.

## INTRODUCTION

Corn is the main energy feed ingredient for animal production [1]. However, corn shortages and distribution difficulties continue unabated due to its high usage in animal and human food, which affects national food security. Since 2020 in China, the National Animal Nutrition Guidance Committee has drawn up several strategies to reduce the utilization of corn and soybean meal in swine and poultry production. One of the strategies emphasized the utilization of paddy rice and its by-products as alternatives to corn. Brown rice contains more energy and protein and has better palatability and nutritional value compared with paddy rice [2,3]. The production of brown rice in China, approximate 2.4 million tons, ranks the first all over the world [2].

Brown rice, known as whole grain rice, consists of 2% to 3% germ, 6% to 7% of bran and 90% of endosperm after removal of the inedible outer hull and is commonly used in animal production [3–6]. However, the great variations in nutrient composition of brown rice have caused a significant difference between the “true value” and the “static parameters” in existing databases due to differences in sources, processing, and storage, making precision formulation difficult. Additionally, the limited studies on the amino acid (AA) digestibility of brown rice also affect the available use of brown rice in diets. Feed formulation can be formulated with precision by dynamically predicting the nutritional composition of ingredients via constructing prediction equations. Therefore, we conducted research to determine the physical and chemical composition, the apparent ileal digestibility (AID) and standardized ileal digestibility (SID) of crude protein (CP) and AA in brown rice fed to pigs and to establish predicted equations for SID_AA_.

## MATERIALS AND METHODS

### Animal care

The animal experiment was carried out in the metabolism laboratory of the Institute of Subtropical Agriculture, Chinese Academy of Sciences (Changsha, China). All the experimental protocol was reviewed and approved by the Animal Care and Use Committee at the Institute of Subtropical Agriculture, Chinese Academy of Sciences (IACUC#201302).

### Sources of brown rice samples

Ten paddy rice samples were collected from Hunan (n = 4), Anhui (n = 1), Hubei (n = 1), Guangxi (n = 1), Henan (n = 1), Jiangxi (n = 1), and Guizhou (n = 1) province and were processed into brown rice after dehulling (Table 1). All brown rice were crushed and sieved through 40-mesh screen and stored at −20°C before chemical analysis [7].

### Animals, diets, and experimental design

A total of 22 pigs (Duroc×[Yorkshire×Landrace], initial body weight: 42.0±1.2 kg) were installed a simple T-cannula in their distal ileum [8]. All pigs were placed in individual metabolism cages (1.4 m×0.7 m×0.5 m) in an environmentally controlled room (23°C±1°C). Pigs were allotted to a replicated 11×3 incomplete Latin square design with 3 consecutive periods and 11 diets. Ten brown rice diets containing the selected brown rice as the sole nitrogen source and a nitrogen-free diet for determination of basal endogenous losses of CP and AA were formulated (Table 2). All diets were supplemented with 0.3% of chromic oxide (Cr_2_O_3_) as an indigestible marker. All diets were fortified with vitamins and minerals to meet or exceed the nutritional requirements recommended by the NRC [9] for 20- to 50-kg pigs. The analyzed CP and AA composition of diets is represented in Table 3.

The diets were provided twice daily (0800 and 1700 h) at the equivalence of 4% of their average initial body weight recorded at the beginning of each period [10], including 5 days of adaptation followed by 2 days of ileal digesta collection [11].

### Sample collection and preparation

Ileal digesta were collected on d 6 and d 7 for 8 h every day from 0800 to 1600 h according to the standard procedure [11]. Cannulas were opened and plastic bags were fastened with the help of a rubber band to collect the digesta flowing into the bags. The bags were replaced every 30 minutes, and the ileal digesta were promptly frozen at −20°C. During the collection, 5 mL of 10% (v/v) formic acid was added into each bag to minimize the bacterial fermentation. At the end of each period, all the digesta samples were thawed, mixed, and lyophilized in a Vacuum-Freeze Dryer (ACIENTZ-50F/A; Ningbo Xinzhi Lyophilization Equipment Co, Ltd, Ningbo, China) for 72 h and subsampled.

### Sample analysis and calculation

The samples of brown rice and diets were analyzed using the procedures for bulk weight (GB 5498-85), thousand-kernel weight (GB/T 5519-88), and thousand-kernel volume (GB/T 5519-88). The AOAC [12] procedures were used to determine the contents of dry matter (DM, 930.15), CP (984.13), ether extract (EE, 920.39), crude ash (Ash, 942.05), calcium (Ca, 968.08), and total phosphorus (TP, 964.06). Total starch (TS) contents were analyzed with a commercial starch assay kit (Megazyme, Bure, Ireland). The contents of crude fiber (CF), neutral detergent fiber (NDF), and acid detergent fiber (ADF) were determined using a fiber analyzer (ANKOM A200i Fiber Analyzer; Beijing ANKOM Technology Co. Ltd, Beijing, China) in combination with fiber bags.

The samples of brown rice, diets and lyophilized ileal digesta were analyzed for the contents of DM, CP, and AA. The fifteen AA profiles were measured by the HPLC (Agilent 1200, Agilent Technologies, Santa Clara, CA, USA) after acid hydrolysis with 6 *M* HCl. Methionine (Met) and cysteine (Cys) were determined after oxidative hydrolysis (method 982.30 E(a); AOAC, 2006). Tryptophan (Trp) was measured after 10% of KOH hydrolysis for 16 to 18 h at 40°C using the spectrophotometric method of GB/T 15400-2018. The AID and SID of AA (%) in brown rice samples were determined using the method of Stein et al [11] described as following:


$$\text{AID}=[1-({\text{AA}}_{\text{d}}\times {\text{T}}_{\text{r}})/({\text{AA}}_{\text{r}}\times {\text{T}}_{\text{d}})]\times 100\%,$$

where AA_d_ and T_d_ represent the concentrations of AA and chromium in the ileal digesta (g/kg of DM), respectively, and AA_r_ and T_r_ are the concentrations of AA and chromium in the brown rice diets (g/kg of DM), respectively. The same equation was used to calculate the AID of CP.


$${\text{IAA}}_{\text{end}}=[{\text{AA}}_{\text{d}}\times ({\text{T}}_{\text{r}}/{\text{T}}_{\text{d}})],$$

where IAA_end_ is the basal endogenous loss of each AA (g/kg of DM intake) and AA_d_ and T_d_ represent the concentrations of AA and chromium in the ileal digesta from the growing-finishing pigs fed the N-free diet, respectively. The T_r_ represents the concentration of chromium in the N-free diet. The same equation was used to calculate the endogenous loss of CP.


$$\text{SID}=[\text{AID}+({\text{IAA}}_{\text{end}}/{\text{AA}}_{\text{d}})\times 100\%].$$

### Statistical analysis

The normality and equal variance of data were assessed using the Descriptive Statistics procedure of SPSS 17.0 (SPSS Inc, Chicago, IL, USA) and outliers were identified by analyzing the Z-scores of the data. Correlation coefficients among the physical characteristics, chemical composition, and AA digestibility (AID and SID of lysine [Lys], Met, Trp, and threonine [Thr]) of brown rice samples were examined using the CORR procedure. The stepwise regression was employed to establish the prediction equations for the SID of Lys, Met, Trp, and Thr of the brown rice samples based on its the physical characteristics and chemical composition. The best-fit equations were selected depending on relative standard deviation (RSD), R^2^, and p-value; p<0.05 means significant difference and p<0.01 means extremely significant difference, when R^2^ is closer to 1 and p-value represent a significant difference, the equation is considered more accurate.

## RESULTS

### Physical characteristics, chemical composition, and AA profile of brown rice

On air-dry basis, the coefficient of variation (CV) of CP, EE, Ash, CF, NDF, ADF, Ca, TP, TS, thousand-kernel weight and thousand-kernel volume were greater than 10%, and the CV of EE, Ca, CF, NDF, and ADF were greater than 30%. The content of CP, EE, Ca, TP, CF, NDF, ADF, TS, thousand-kernel weight and thousand-kernel volume in 10 brown rices averaged 6.73% (5.34% to 8.39%), 1.97% (1.10% to 2.77%), 0.02% (0.01% to 0.03%), 0.17% (0.14% to 0.20%), 0.92% (0.20% to 1.53%), 5.04% (2.17% to 9.58%), 1.61% (0.74% to 2.49%), 69.08% (56.76% to 77.94%), 20.53 g/kg kernel (14.59 to 25.82 g/kg kernel) and 25.22 mL/1,000 grain (19.90 to 32.00 mL/1,000 grain), respectively (Table 4). The CV of all AA contents exceeded 10%. The concentrations of Lys, Met, Thr, and Trp in 10 brown rice samples were 0.34% (0.25% to 0.49%), 0.14% (0.07% to 0.26%), 0.24% (0.19% to 0.30%), and 0.07% (0.06% to 0.10%), respectively.

### AID and SID of CP and AA

As shown in Table 5, the AID_CP_ wa6-181s 37.8% (20.0% to 54.6%). The AID_Lys_, AID_Met_, AID_Thr_, and AID_Trp_ were 63.7% (49.7% to 73.9%), 73.9% (57.0% to 87.0%), 36.1% (25.4% to 47.0%), and 76.2% (68.6% to 81.3%), respectively. The SID of CP and AA of 10 brown rices were shown in Table 6. The SID_CP_ was 77.2% (62.6% to 85.5%), and the SID of Lys, Met, Thr, and Trp ranged from 80.3% to 94.3% (87.5%), 78.9% to 98.9% (89.2%), 46.1% to 67.6% (55.4%), and 86.3% to 96.3% (92.5%), respectively.

### Correlation analysis and prediction equations for SID of CP and AA

The correlation among physical characteristics, chemical composition, and the SID of the first four limiting AA of brown rice is presented in Table 7. The SID of Thr was positively related to EE (p<0.05). As shown in Table 8, the best fit equations for SID_CP_, SID_Lys_, SID_Thr_, and SID_Trp_ as following, SID_CP_ = −664.181+8.484×DM (R^2^ = 0.40; RSD = 6.46; p = 0.05), SID_Lys_ = 53.126+6.031×EE+0.893×thousand-kernel volume (R^2^ = 0.66; RSD = 3.43; p<0.05), SID_Thr_ = 39.916+ 7.843×EE (R^2^ = 0.41; RSD = 5.99; p<0.05) and SID_Trp_ = −361.588+4.891×DM+0.387×TS (R^2^ = 0.85; RSD = 1.66; p<0.01).

## DISCUSSION

### Physical characteristics, chemical composition, and AA profile of brown rice

Great variation in chemical composition and physical property was observed among 10 brown rice samples. The CV value for CP, Ash, TP, TS, 1,000 kernel weight and 1,000 kernel volume exceeded 10%, and EE, Ca, CF, NDF and ADF even surpassed 30%. The detected contents of gross energy (GE), DM, EE, Ca, and TS were within the range of the tabulated value [13,14], and most chemical compositions and physical properties were close to the values in previous literature [15–19], indicating that our results were credible. However, bulk weight, 1,000 kernel weight and 1,000 kernel volume were not provided in tabulated value, the CP, Ash, and TP concentrations were lower than the tabulated value, and the content of CF, NDF, and ADF were higher than the tabulated value, which may be attributed to the incomplete shelling of paddy rice to obtain brown rice. This phenomenon was confirmed by the findings that a large number of hulls were not completely removed due to the inefficiency of sheller and the analyzed fiber (CF, NDF, and ADF) contents were relatively high in our study. The separation of different parts of grains during the milling process, might influence the physical and chemical properties of rice by-products [20]. Chen [21] pointed out that the quality of paddy rice is mainly related to varieties, starch content, storage, and environment. The nutrients of brown rice in this study are inconsistent with published data, which may be due to differences in paddy rice cultivation regions, periods, and environments. In addition, the variation of brown rice might result from processing and storage technology and different growth conditions, such as climate and soil conditions [22,23].

The AA and protein contents of brown rice varied greatly. The 10 brown rice samples were from different regions with multiple natural conditions in China, which provided an explanation for the deviation from uniformity. The analyzed CP and AA values in our study were close to the reported values in the database [16,24,25]. Lysine, Met, Thr, and Trp are the main limiting AAs in livestock and poultry, and play an irreplaceable role in pig growth [26]. He et al [27] analyzed and compared the AA content of 18 varieties of brown rice samples and found that the first, second and third limiting AA were Lys, Thr, and Ile when brown rice used in corn-brown rice-SBM fed to pigs. In our study, the content of Lys, Thr, and Ile in brown rice were similar to tabulated value [13,14].

### SID of AA in brown rice

Diversified low-protein diets for swine production have been fully pushing ahead in China [28]. Accuracy estimation of AA availability in diets or feedstuffs is the basis for the comprehensive implementation of low-protein diets system. Standardized ileal digestibility of AA is recognized as the gold method for estimation of AA availability [11]. Lysine, Met, Thr, and Trp are the main limiting AA in poultry. In the study, the mean SID values of the Lys, Met, and Trp in brown rice samples were greater than those in the Nutrient Requirements of Swine in China [11]. Meanwhile, the analyzed SID of the Lys, Met, and Trp contents fell within the range of the reports by Li [24], Wu et al [25], and Zhang et al [29].

### Correlation analysis and prediction equations for SID of AA in brown rice

The SID was calculated by correcting AID for the ileal basal endogenous losses and dietary composition, especially dietary protein, and fiber, is responsible for ileal basal endogenous CP and AA losses [30,31]. In the current study, the SID_Thr_ was positively correlated with EE. The increase in dietary fat delayed gastric emptying [32], and the slower gastric emptying may result in slower rate of passage of the diet, causing an increase in the time of exposure of feed to proteolytic enzymes, thus providing longer time for peptides and AA to be digested and absorbed, and increase in AID of AA [33,34]. The addition of oil to diets fed to growing pigs increased not only the AID but also the SID of AA [35,36]. Imbeah and Sauer [37] concluded that the level of fat may affect ileal AA digestibility. Additionally, positive correlation trends between SID_Lys_ or SID_Thr_ and EE, and SID_Trp_ with DM and TS were observed. The release rate of glucose during the digestion process of feed starch from different sources varies, and the synchronization degree of glucose and AA supply varies. Therefore, the AA absorption of feed starch from different sources are also different [38]. The digestibility of starch also directly affects the absorption of AA in the intestine [38]. Also, we obtained that DM and GE are closely related in brown rice, and with the raise of DM, the GE and TP will also increase, and then affect the SID of AA [39]. Unfortunately, there is no literature specifically explaining the interaction between DM and SID_AA_, nor the mechanism by which DM has a negative impact on CP and AA digestibility, which may be one of the questions that we need to explore in our subsequent experiments. Our findings suggest that EE, DM, and TS might be key predictors for SID of Lys, Met, Thr, and Trp.

The research on a prediction model of AA digestibility in brown rice for pigs is limited. Nutritional Requirements of Chinese Pigs [13] reported that CP was the key predictors to estimate the SID of the first four limiting AA in brown rice. Yu et al [40] found that there is no correlation between biomimetic digestion and biological measurements of Thr, histidine, arginine, and Cys. However, Liu et al [41] established a prediction equation for SID_Lys_, SID_Met_, and SID_Thr_ in sunflower seeds, showed a positive correlation between SID_Lys_ and Met, and a negative correlation between SID_Lys_ and Trp, the SID_Met_ was negatively correlated with EE and positively with Ca and Met, the SID_Thr_ was positive with Met. Yun et al [42] established a prediction equation for SID_Lys_ and SID_Met_ in wheat, showing a positive correlation between SID_Lys_, SID_Met_, and NDF. In the present study, we selected 10 brown rice to do a similar study and got one prediction equation for SID_CP_, two equations for SID_Lys_, one equation for SID_Thr_ and two equations for SID_Trp_, respectively. Meanwhile, the key predictors for the SID of CP and AA are DM, EE, 1,000-kernel volume, and TS. Until now, the prediction equations of SID of AA are not as applicable as the available energy in practice due to the complex factors, mainly including accurate assessment of endogenous nitrogen losses and determination of AA, and more effort and work are required for the future.

## CONCLUSION

In summary, the physicochemical properties of 10 brown rice showed a huge difference. The SID of CP and the first four limiting AA could be estimated from the analyzed contents of EE, DM, 1,000-kernel volume and total starch.

## Figures and Tables

**Table 1:** Sources of brown rice

Sample numbers	Sources
BR1	Yueyang city, Hunan
BR2	Guangde city, Anhui
BR3	Zhijiang city, Hubei
BR4	Liuzhou city, Guangxi
BR5	Zhumadian city, Henan
BR6	Jishou city, Hunan
BR7	Yongzhou city, Hunan
BR8	Jingde city, Jiangxi
BR9	Qiannan prefecture, Guizhou
BR10	Changsha city, Hunan

BR, Brown rice.

**Table 2:** Ingredients composition of experimental diet and nitrogen-free diet (air-dry basis, %)

Items	Experimental diet	Nitrogen-free diet
Corn starch	42.90	78.90
Brown rice	40.00	-
Soybean oil	3.00	3.00
Cellulose acetate	-	4.00
Sucrose	10.00	10.00
Limestone	0.50	0.50
Calcium phosphite (Ca(H_2_PO_4_)_2_)	1.90	1.90
Chromium oxide (Cr_2_O_3_)	0.30	0.30
Sodium chloride (NaCl)	0.40	0.40
Potassium carbonate (K_2_CO_3_)	0.40	0.40
Magnesium oxide (MgO)	0.10	0.10
Vitamin and mineral premix^1)^	0.50	0.50
Total	100.00	100.00

1) The vitamin and mineral premix provided the following per kg of diets: vitamin A 4,200 IU, vitamin D_3_ 400 IU, vitamin E 36 IU, vitamin K_3_ 1.2 mg, vitamin B_12_ 23 μg, vitamin B_2_ 5.63 mg, vitamin B_5_ 20.5 mg, vitamin B_3_ 28 mg, choline chloride 1.00 g, folic acid 0.8 mg, vitamin B_1_ 3.4 mg, vitamin B_6_ 2.7 mg, vitamin H 0.18 mg, Mn (as manganese sulfate) 40.0 mg, Fe (as ferrous sulfate) 70.0 mg, Zn (as copper sulfate) 70 mg, I (as potassium iodide) 0.3 mg, Se (as sodium selenite) 0.3 mg.

**Table 3:** Analyzed chemical composition of experiment diets (air-dry basis, %)

Items	Brown rice diet										Mean	CV (%)	N-free diet

	1	2	3	4	5	6	7	8	9	10			
DM (%)	89.71	89.46	89.23	88.84	89.29	89.46	89.11	89.28	89.52	89.57	89.35	0.281	90.21
CP (%)	3.01	2.59	3.31	4.24	4.60	4.35	4.96	2.80	3.07	2.80	3.57	24.39	0.51
Essential amino acids (%)													
Arginine	0.21	0.21	0.20	0.27	0.37	0.33	0.35	0.19	0.22	0.21	0.26	27.13	0.03
Histidine	0.10	0.11	0.10	0.13	0.18	0.17	0.17	0.10	0.10	0.09	0.12	28.03	-
Isoleucine	0.11	0.12	0.13	0.15	0.21	0.19	0.20	0.12	0.12	0.11	0.15	26.76	-
Leucine	0.25	0.24	0.23	0.32	0.41	0.39	0.39	0.23	0.26	0.24	0.30	24.90	0.03
Lysine	0.15	0.15	0.15	0.21	0.30	0.30	0.32	0.14	0.15	0.14	0.20	36.56	0.03
Methionine	0.02	0.07	0.06	0.04	0.08	0.08	0.06	0.05	0.03	0.06	0.05	39.03	-
Phenylalanine	0.16	0.17	0.15	0.23	0.28	0.26	0.27	0.17	0.18	0.14	0.20	26.78	0.02
Threonine	0.10	0.12	0.10	0.14	0.18	0.17	0.18	0.09	0.11	0.10	0.13	27.91	0.03
Tryptophan	0.05	0.05	0.04	0.05	0.06	0.06	0.07	0.05	0.04	0.04	0.05	19.69	0.02
Valine	0.16	0.15	0.16	0.21	0.26	0.25	0.24	0.15	0.17	0.15	0.19	23.68	0.02
Non-essential amino acids (%)													
Alanine	0.18	0.18	0.18	0.23	0.27	0.26	0.25	0.17	0.18	0.17	0.21	18.85	0.03
Aspartate	0.29	0.28	0.27	0.38	0.50	0.47	0.51	0.26	0.31	0.28	0.36	28.69	0.03
Cystine	0.02	0.02	0.01	0.05	0.06	0.06	0.06	0.02	0.02	0.02	0.03	60.82	-
Glutamine	0.60	0.59	0.52	0.78	0.99	0.94	0.95	0.55	0.67	0.58	0.72	25.58	0.06
Glycine	0.14	0.14	0.13	0.18	0.23	0.22	0.22	0.13	0.15	0.14	0.17	24.17	0.02
Proline	0.18	0.17	0.16	0.21	0.31	0.29	0.31	0.18	0.23	0.18	0.22	27.14	0.06
Serine	0.13	0.12	0.09	0.18	0.20	0.19	0.20	0.10	0.14	0.12	0.15	28.32	0.02
Tyrosine	0.06	0.07	0.05	0.08	0.12	0.10	0.08	0.05	0.07	0.07	0.08	28.26	0.03

CV, coefficient of variation; DM, dry matter; CP, crude protein.

**Table 4:** Analyzed chemical composition, physical characteristics of 10 brown rice samples (air-dry basis, %)

Items	Brown rice number										Mean	CV (%)

	BR1	BR2	BR3	BR4	BR5	BR6	BR7	BR8	BR9	BR10		
DM	88.40	87.22	86.82	87.75	87.11	87.99	87.52	87.26	86.40	87.42	87.39	0.66
GE (MJ/kg)	15.90	15.18	15.15	15.95	15.37	15.64	15.71	15.28	15.07	14.98	15.42	2.28
CP	6.63	6.30	5.34	8.39	8.05	5.90	6.37	7.11	6.64	6.55	6.73	13.68
EE	2.62	2.41	1.10	2.08	1.50	1.65	1.32	2.77	1.64	2.59	1.97	30.63
Ash	1.22	1.09	1.05	1.00	1.29	1.14	0.95	1.13	0.87	0.87	1.06	13.41
CF	0.85	0.82	0.60	1.26	1.19	0.90	0.98	1.53	0.85	0.20	0.92	39.90
NDF	4.46	4.00	4.49	5.00	4.22	5.09	7.21	9.58	4.17	2.17	5.04	40.05
ADF	1.71	1.87	1.46	1.09	1.76	2.08	1.04	2.49	1.82	0.74	1.61	32.85
Ca	0.02	0.01	0.01	0.01	0.02	0.03	0.01	0.02	0.01	0.01	0.02	34.76
TP	0.20	0.19	0.17	0.17	0.17	0.17	0.19	0.16	0.14	0.16	0.17	11.39
Total starch	69.38	69.01	75.01	58.36	56.76	69.00	66.22	76.38	72.77	77.94	69.08	10.30
Bulk weight (kg/L)	0.77	0.73	0.76	0.75	0.76	0.77	0.76	0.78	0.77	0.75	0.76	1.73
Thousand -kernel weight (g/kg kernel)	18.3	21.4	21.0	14.6	18.8	24.4	19.7	20.2	25.8	21.0	20.5	15.3
Thousand -kernel volume (mL/1,000 grain)	22.6	26.0	27.0	19.9	24.7	32.0	25.9	24.5	22.1	27.5	25.2	13.3
Essential amino acids (%)												
Arginine	0.57	0.40	0.44	0.55	0.57	0.47	0.55	0.45	0.45	0.39	0.48	14.28
Histidine	0.25	0.21	0.22	0.26	0.27	0.22	0.34	0.20	0.21	0.17	0.23	20.47
Isoleucine	0.31	0.24	0.27	0.30	0.32	0.25	0.63	0.25	0.24	0.21	0.30	39.66
Leucine	0.60	0.47	0.52	0.62	0.65	0.52	0.11	0.49	0.53	0.44	0.50	30.35
Lysine	0.35	0.29	0.31	0.35	0.38	0.34	0.49	0.32	0.28	0.25	0.34	19.77
Methionine	0.14	0.10	0.12	0.12	0.16	0.15	0.26	0.13	0.07	0.11	0.14	37.30
Phenylalanine	0.44	0.33	0.39	0.44	0.46	0.37	0.43	0.35	0.37	0.31	0.39	13.27
Threonine	0.26	0.20	0.23	0.26	0.28	0.24	0.30	0.21	0.22	0.19	0.24	15.67
Tryptophan	0.06	0.07	0.07	0.08	0.10	0.07	0.08	0.07	0.06	0.08	0.07	16.60
Valine	0.41	0.32	0.36	0.42	0.43	0.36	0.49	0.34	0.35	0.32	0.38	14.37
Non-essential amino acids (%)												
Alanine	0.43	0.34	0.38	0.44	0.46	0.37	0.49	0.37	0.37	0.34	0.40	12.57
Aspartate	0.68	0.53	0.60	0.69	0.74	0.60	0.97	0.59	0.58	0.50	0.65	20.80
Cystine	0.10	0.07	0.10	0.07	0.04	0.13	0.07	0.09	0.09	0.09	0.08	28.65
Glutamine	1.38	1.10	1.21	1.46	1.50	1.21	1.62	1.10	1.23	0.98	1.28	15.97
Glycine	0.35	0.28	0.30	0.34	0.37	0.30	0.39	0.29	0.29	0.24	0.32	14.45
Proline	0.36	0.29	0.34	0.39	0.37	0.33	0.40	0.32	0.30	0.26	0.34	13.72
Serine	0.31	0.28	0.27	0.38	0.34	0.28	0.30	0.25	0.30	0.22	0.29	15.57
Tyrosine	0.19	0.12	0.10	0.19	0.17	0.16	0.08	0.16	0.11	0.13	0.14	26.70

BR, brown rice; CV, coefficient of variation; DM, dry matter; GE, gross energy; CP, crude protein; EE, ether extract; Ash, crude ash; CF, crude fiber; NDF, neutral detergent fiber; ADF, acid detergent fiber; Ca, calcium; TP, total phosphorus.

**Table 5:** Apparent ileal digestibility of crude protein (CP) and amino acids in brown rice fed to growing-finishing pigs (%)

Items	Brown rice										SEM	p-value

	BR1	BR2	BR3	BR4	BR5	BR6	BR7	BR8	BR9	BR10		
CP (%)	33.1^bc^	32.7^b^	33.0^bc^	32.8^a^	34.0^bc^	51.7^a^	54.6^a^	30.0^bc^	20.0^c^	30.9^bc^	3.71	<0.001
Essential amino acids (%)												
Arginine	38.8^abc^	31.6^bc^	26.0^c^	40.7^ab^	35.5^abc^	39.8^abc^	39.9^a^	38.4^abc^	31.0^bc^	39.6^abc^	1.50	0.044
Histidine	68.3^bc^	67.4^bcd^	61.2^de^	69.7^ab^	73.9^ab^	80.8^a^	78.5^ab^	64.8^cd^	56.3^e^	67.8^bcd^	2.24	<0.001
Isoleucine	63.4^d^	73.5^ab^	66.8^bc^	75.0^a^	73.4^ab^	78.5^a^	76.9^ab^	72.1^ab^	64.4^cd^	73.9^ab^	1.55	<0.001
Leucine	77.9^bcd^	77.7^abc^	72.4^d^	79.0^a^	78.0^abc^	82.8^a^	79.9^ab^	77.1^abcd^	74.4^cd^	81.3^a^	0.92	<0.001
Lysine	63.1^cd^	60.2^cd^	52.5^de^	64.9^abc^	67.0^abc^	73.9^a^	73.8^a^	63.4^bc^	49.7^e^	68.1^abc^	2.39	<0.001
Methionine	59.1^c^	87.0^a^	77.5^ab^	71.6^b^	78.1^ab^	81.3^ab^	69.6^b^	70.9^b^	57.0^c^	87.0^a^	3.11	<0.001
Phenylalanine	79.4^bc^	82.4^abc^	75.9^bc^	80.6^bc^	79.3^abc^	85.5^ac^	84.1^a^	80.3^ab^	77.3^bc^	79.1^ab^	0.89	<0.001
Threonine	36.4^b^	32.1^b^	25.4^b^	32.3^ab^	29.8^ab^	27.4^b^	44.4^ab^	45.2^ab^	40.7^ab^	47.0^a^	2.35	0.042
Tryptophan	79.5^ab^	72.3^c^	74.2^c^	74.1^bc^	71.8^c^	80.9^a^	81.3^a^	80.6^a^	68.6^d^	78.8^ab^	1.35	<0.001
Valine	65.6^d^	66.8^abc^	64.5b^cd^	76.2^a^	71.6^abc^	77.7^ab^	75.4^ab^	68.7^bcd^	65.5^cd^	73.8^ab^	1.49	<0.001
Non-essential amino acids (%)												
Alanine	52.4^abc^	41.8^bc^	44.0^c^	56.2^a^	43.2^c^	56.7^a^	56.6^a^	51.0^abc^	36.4^c^	57.2^ab^	2.28	<0.001
Aspartate	61.9^cd^	63.1^de^	56.0^e^	66.5^ab^	64.9^abcd^	72.3^a^	72.0^abc^	62.7^bcd^	54.9^de^	69.7^abc^	1.80	<0.001
Cystine	43.0	29.5	40.3	46.3	27.8	52.7	46.8	44.2	28.3	55.6	2.98	0.066
Glutamine	77.0^bc^	74.7^bc^	71.4^d^	77.5^a^	74.5^bc^	80.5^a^	79.6^abc^	77.1^abc^	75.3^c^	79.8^ab^	0.85	<0.001
Glycine	54.0^b^	47.6^c^	42.9^bc^	70.7^a^	56.5^b^	74.9^a^	75.1^a^	48.3^bc^	41.4^bc^	54.4^b^	3.82	<0.001
Proline	41.6^bc^	38.7^c^	34.0^c^	54.6^ab^	38.9^bc^	67.3^a^	58.6^a^	60.7^bc^	31.4^c^	42.8^bc^	3.56	<0.001
Serine	52.0^bc^	38.9^cd^	20.3^e^	59.8^ab^	51.8^bc^	67.9^a^	59.0^ab^	37.1^d^	39.0^cd^	54.2^ab^	4.21	<0.001
Tyrosine	38.0^bc^	51.9^ab^	37.7^bc^	31.7^c^	35.2^bc^	41.8^bc^	31.0^c^	47.2^abc^	49.1^bc^	62.8^a^	3.02	0.015

BR, brown rice; SEM, standard error of the mean.

a–e Means in the same row with common letters are not different at p<0.05.

**Table 6:** Standardlized ileal digestibility (SID) of crude protein (CP) and amino acids (AA) in brown rice fed to growing-finishing pigs (%)

Items	Brown rice										SEM	p-value

	BR1	BR2	BR3	BR4	BR5	BR6	BR7	BR8	BR9	BR10		
CP (%)	78.4^ab^	85.3^a^	73.9^bc^	77.7^ab^	65.7^c^	85.5^a^	83.9^ab^	79.4^ab^	62.6^c^	79.5^ab^	2.34	<0.001
Essential amino acids (%)												
Arginine	86.1	77.3	72.0	82.3	70.6	78.6	79.1	90.1	75.4	79.5	1.79	0.125
Histidine	84.5^a^	86.7^ab^	82.4^bc^	85.9^a^	88.5^ab^	93.3^a^	90.7^ab^	86.2^ab^	76.2^c^	89.7^ab^	1.43	<0.001
Isoleucine	83.8^d^	95.0^c^	87.8^bcd^	92.7^ab^	85.9^cd^	92.9^abc^	90.3^abc^	94.0^abc^	86.7^d^	93.0^a^	1.17	<0.001
Leucine	93.1^bcd^	93.4^abcd^	87.3^d^	90.9^ab^	87.3^cd^	92.6^abc^	89.6^abcd^	93.5^abcd^	88.8^d^	93.3^a^	0.77	<0.001
Lysine	84.3^abc^	90.7^abc^	81.2^cd^	87.6^ab^	82.6^bcd^	92.5^abc^	88.5^abc^	94.3^a^	80.3^d^	93.1^a^	1.54	<0.001
Methionine	78.9^b^	96.7^a^	89.0^ab^	88.9^ab^	86.2^ab^	89.9^ab^	86.5^b^	94.0^ab^	83.1^b^	98.9^a^	1.83	0.024
Phenylalanine	93.4	98.1	91.4	91.9	90.7	95.7	93.7	95.7	91.5	94.1	0.71	0.079
Threonine	56.5^bc^	49.7^bc^	46.1^c^	50.6^bc^	48.6^bc^	53.2^c^	55.5^abc^	67.6^ab^	58.5^abc^	67.1^a^	2.21	0.014
Tryptophan	95.7^a^	89.9^bc^	94.2^ab^	91.1^ab^	86.3^c^	96.3^a^	92.9^ab^	96.0^a^	87.2^c^	95.8^a^	1.13	<0.01
Valine	86.5^c^	90.4^ab^	87.5^abc^	93.4^a^	85.5^bc^	92.6^ab^	90.2^abc^	92.7^abc^	87.3^c^	91.6^a^	0.86	0.010
Non-essential amino acids (%)												
Alanine	95.2^ab^	84.0^abc^	85.7^bc^	94.0^ab^	71.0^d^	90.0^abc^	89.4^abc^	94.0^ab^	76.6^cd^	100.2^a^	2.68	<0.001
Aspartate	84.7^abcd^	86.9^abcd^	82.6^cd^	89.4^ab^	81.9^d^	90.5^abc^	88.7^bcd^	95.2^ab^	84.9^cd^	94.4^a^	1.38	<0.001
Cystine	62.1	55.8	62.5	73.5	69.5	65.0	64.2	77.5	65.9	79.6	2.23	0.277
Glutamine	92.7^bc^	92.5^bc^	87.2^cd^	91.0^ab^	87.3^d^	91.8^abc^	90.6^bcd^	96.4^ab^	90.9^bcd^	95.9^a^	0.91	<0.001
Glycine	74.1^abc^	66.9^bc^	63.7^c^	86.1^a^	68.6^bc^	87.8^a^	87.7^a^	69.9^bc^	60.4^bc^	74.8^ab^	3.02	<0.001
Proline	70.2^ab^	71.1^ab^	71.1^ab^	79.3^a^	75.1^b^	84.9^a^	84.4^a^	79.2^ab^	58.0^b^	72.0^ab^	2.39	0.013
Serine	89.4^a^	88.5^abc^	80.7^c^	87.7^a^	81.9^bc^	96.7^a^	88.2^abc^	95.9^ab^	82.8^c^	93.6^a^	1.69	<0.001
Tyrosine	68.9^bcd^	84.6^ab^	74.8^abcd^	55.1^d^	65.7^d^	62.2^cd^	55.4^d^	86.9^abc^	72.4^bcd^	90.5^a^	3.79	<0.001

BR, brown rice; SEM, standard error of the mean.

Values for SID were calculated by correcting the apparent ileal digestibility values with the basal endogenous losses (IAA end). IAA end (g/kg dry matter intake) averaged as CP, 16.72; Arg, 1.11; His, 0.24; Ile, 0.31; Leu, 0.44; Lys, 0.53; Met, 0.08; Phe, 0.30; Thr, 0.24; Trp, 0.09; Val, 0.42; Ala, 0.86; Asp, 0.98; Cys, 0.08; Glu,1.21; Gly, 0.32; Pro, 0.59; Ser, 0.69; Tyr, 0.22.

a–e Means in the same row with common letter, are not different at p<0.05.

**Table 7:** Correlation coefficients (r) among physical characteristics, chemical constituents and the standardlized ileal digestibility (SID) of the first four limiting amino acids of the 10 brown rice samples

Items	SID_CP_^1)^	SID_Lys_	SID_Met_	SID_Thr_	SID_Trp_	Gross energy	Dry matter	Crude protein	Ether extract	Crude ash	Crude fiber	Neutral detergent fiber	Acid detergent fiber	Calcium	Total phosphorus	Total starch	Bulk weight	Thousand -kernel weight
SIDLys	0.774^**^																	
SIDMet	0.452	0.723^*^																
SIDThr	0.099	0.538	0.297															
SIDTrp	0.664^*^	0.579	0.236	0.433														
Gross energy	0.164	−0.025	−0.452	−0.273	0.167													
Dry matter	0.624	0.402	−0.163	0.051	0.582	0.743^*^												
Crude protein	−0.325	−0.021	−0.108	0.040	−0.454	0.425	0.101											
Ether extract	0.322	0.578	0.349	0.641^*^	0.361	0.197	0.391	0.248										
Crude ash	0.020	−0.067	−0.272	−0.356	−0.008	0.499	0.390	0.206	0.076									
Crude fiber	0.215	0.219	−0.244	−0.078	−0.020	0.352	0.239	0.302	−0.177	0.343								
Neutral detergent fiber	0.235	0.315	−0.053	0.262	0.250	0.072	0.059	0.097	0.067	0.191	0.794^**^							
Acid detergent fiber	−0.062	0.083	−0.104	0.044	−0.014	0.119	−0.083	−0.067	0.174	0.564	0.430	0.494						
Calcium	0.170	0.260	−0.187	0.049	0.331	0.455	0.495	−0.009	0.091	0.686^*^	0.463	0.252	0.621					
Total phosphorus	0.590	0.097	−0.183	−0.352	0.244	0.388	0.667^*^	−0.124	0.116	0.454	0.141	0.046	−0.114	0.090				
Total starch	0.159	0.274	0.364	0.602	0.565	−0.536	−0.224	−0.685^*^	0.301	−0.418	−0.456	0.042	0.116	−0.108	−0.287			
Bulk weight	−0.281	−0.110	−0.491	0.388	0.306	0.159	0.066	−0.038	−0.059	0.195	0.405	0.533	0.468	0.556	−0.314	0.227		
Thousand -kernel weight	−0.137	−0.030	0.072	0.181	−0.027	−0.553	−0.451	−0.641^*^	−0.221	−0.278	−0.158	−0.128	0.364	0.174	−0.458	0.554	0.229	
Thousand -kernel volume	0.470	0.426	0.415	−0.009	0.449	−0.309	0.107	−0.660^*^	−0.220	0.085	−0.018	−0.081	0.118	0.455	0.054	0.345	0.007	0.535

1) CP, crude protein; AA, amino acid; Lys, Lysine; Thr, Threonine; Met, methionine; Trp, Tryptophan; SID_CP_, SID_Lys_, SID_Met_, SID_Thr_, and SID_Trp_, SID of CP, Lys, Met, Thr, and Trp, respectively.

* Means significant difference (p<0.05);

** means extremely significant difference (p<0.01).

**Table 8:** Stepwise regression equations for SID of CP, Lys, Met, Thr, and Trp based upon the chemical characteristics of the 10 brown rice samples (air-dry basis, %)

Items	Prediction equations	RSD	R^2^	p-value
SID_CP_	SID_CP_ = −664.181+8.484×DM	6.46	0.40	0.05
SID_Lys_	SID_Lys_ =77.794+4.939×EE	4.46	0.33	0.08
SID_Lys_	SID_Lys_ = 53.126+6.031×EE+0.893×Thousand-kernel volume	3.43	0.66	<0.05
SID_Thr_	SID_Thr_ = 39.916+7.843×EE	5.99	0.41	<0.05
SID_Trp_	SID_Trp_ = −240.795+3.814×DM	3.24	0.34	0.08
SID_Trp_	SID_Trp_ = −361.588+4.891×DM+0.387×Total starch	1.66	0.85	<0.01

SID, standardized ileal digestibility; CP, crude protein; Lys, Lysine; Thr, Threonine; Trp, Tryptophan; RSD, relative standard deviation; R^2^, R-square; DM, dry matter; EE, ether extract.

p<0.05 means significant difference; p<0.01 means extremely significant difference.
